# Surfactant protein A as a biomarker of outcomes of anti-fibrotic drug therapy in patients with idiopathic pulmonary fibrosis

**DOI:** 10.1186/s12890-020-1060-y

**Published:** 2020-01-31

**Authors:** Takumi Yoshikawa, Mitsuo Otsuka, Hirofumi Chiba, Kimiyuki Ikeda, Yuki Mori, Yasuaki Umeda, Hirotaka Nishikiori, Koji Kuronuma, Hiroki Takahashi

**Affiliations:** Department of Respiratory Medicine and Allergology, Sapporo Medical University School of Medicine, 1-37, South 1-West 16, Chuo-ku, Sapporo, Hokkaido 060-8543 Japan

**Keywords:** Surfactant protein-A (SP-A), Surfactant protein-D (SP-D), Krebs von den Lungen-6 (KL-6), Idiopathic pulmonary fibrosis (IPF), Pirfenidone, Nintedanib

## Abstract

**Background:**

Idiopathic pulmonary fibrosis (IPF) is a progressive and fibrosing lung disease with poor prognosis. Pirfenidone and nintedanib are anti-fibrotic drugs used for patients with IPF. These drugs reduce the rate of decline in forced vital capacity (FVC). Serum surfactant protein (SP)-A, SP-D, and Krebs von den Lungen-6 (KL-6) are monitoring and prognostic biomarkers in patients with IPF; however, their relationship with the therapeutic outcomes of anti-fibrotic drugs has not been investigated. We aim to clarify whether serum SP-A, SP-D, and KL-6 reflect therapeutic outcomes of pirfenidone and nintedanib administration in patients with IPF.

**Methods:**

We retrospectively investigated patients with IPF who were initiated on pirfenidone or nintedanib administration between January 2014 and June 2018 at our hospital. Changes in clinical parameters and serum SP-A, SP-D, and KL-6 levels were evaluated. Patients with ≥10% decline in FVC or ≥ 15% decline in diffusing capacity of the lung for carbon monoxide (DLco) from baseline to 6 months were classified as progression group, while the other patients were classified as stable group.

**Results:**

Forty-nine patients were included (pirfenidone, 23; nintedanib, 26). Stable group comprised 32 patients, while progression group comprised 17 patients. In the stable group, changes in SP-A and KL-6 from baseline to 3 and 6 months significantly decreased compared with the progression group (SP-A: 3 months − 6.0% vs 16.7%, 6 months − 10.2% vs 20.2%, KL-6: 3 months − 9.2% vs 6.7%, 6 months − 15.0% vs 12.1%, *p* < 0.05). Changes in SP-A and SP-D levels showed significant negative correlations with the change in %FVC (r = − 0.46 and r = − 0.39, *p* < 0.01, respectively) and %DLco (r = − 0.67 and r = − 0.54, p < 0.01, respectively). Similar results were also seen in subgroup analysis for both pirfenidone and nintedanib groups. On logistic regression analysis, decrease in SP-A from baseline to 3 months and 6 months was found to predict the outcomes at 6 months (odds ratios: 0.89 and 0.88, respectively).

**Conclusions:**

Changes in serum SP-A reflected the outcomes of anti-fibrotic drug therapy. Serum SP-A has a potential as a biomarker of therapeutic outcomes of anti-fibrotic drugs.

## Background

Idiopathic pulmonary fibrosis (IPF) is a chronic, progressive fibrosing interstitial lung disease of unknown etiology [1]. Patients with IPF have a poor prognosis. The clinical course is typically characterized by acute exacerbations, progression to respiratory failure, and a high risk of lung cancer. The reported median survival of these patients is 3–5 years [2, 3]. Lung transplantation is the only curative treatment for IPF. However, notable advances have recently been made in pharmacological therapy (pirfenidone and nintedanib) for patients with IPF [4, 5].

Pirfenidone and nintedanib were shown to exhibit both anti-fibrotic and anti-inflammatory effects in patients with IPF; these were shown to reduce the rate of decline in forced vital capacity (FVC) and ameliorate disease progression [4, 5]. Pirfenidone has several anti-inflammatory and anti-fibrotic effects, including inhibition of collagen synthesis, down-regulation of transforming growth factor beta (TGF-β), and reduction in fibroblast proliferation [6]. Nintedanib is an intracellular inhibitor of tyrosine kinase that inhibits vascular endothelial growth factor (VEGF), fibroblast growth factors (FGFs), and platelet-derived growth factors (PDGFs) receptors [5]. Consequently, the ATS/ERS/JRS/ALAT statement conditionally recommends the use of pirfenidone and nintedanib in patients with IPF [7].

Progression of IPF is monitored by respiratory symptoms, pulmonary function tests (PFTs), and high-resolution computed tomography (HRCT) [8]. Of these parameters, PFTs are the most important methods for objective monitoring and quantification of disease progression. In particular, decline in FVC and diffusion capacity of the lung for carbon monoxide (DLco) are surrogate markers for clinical monitoring and prognostic assessment of patients with IPF [9]. In ASCEND and INPULSIS-1/2 trials, the primary endpoint was the annual rate of decline in FVC [4, 5]. Amelioration of the decline in FVC and DLco over time is the only indicator for evaluation of therapeutic effect. Therefore, it is desirable to develop other biomarkers for assessment and prediction of therapeutic efficacy.

Surfactant protein (SP)-A and SP-D are secretory proteins that belong to the collectin family, while Krebs von den Lungen (KL)-6 is a mucin-like high-molecular-weight glycoprotein classified as MUC-1 mucin. These are produced mainly by alveolar type II cells [10, 11]. These have been widely proved as serum biomarkers for the diagnosis of interstitial lung disease [10–12]; in addition, these were shown to be related to progression of IPF and the associated mortality [12–14]. However, the relationship between the changes in these serum biomarkers and the effects of pirfenidone and nintedanib in patients with IPF has not been reported. The purpose of this study was to assess whether these serum biomarkers reflect the changes in FVC and DLco, and could serve as biomarkers to monitor the therapeutic outcomes of pirfenidone and nintedanib in patients with IPF.

## Methods

### Subjects

We performed a retrospective cohort study of consecutive patients with IPF who were treated at Sapporo Medical University Hospital. This study was approved by the Institutional Review Board of Sapporo Medical University Hospital (#302–120, dated November 8, 2018). The requirement for written informed consent of patients was waived off because of the retrospective nature of the study. IPF was diagnosed according to the ATS/ERS/JRS/ALAT IPF statement 2018 [1]. Ninety-three patients with IPF were newly initiated on pirfenidone or nintedanib therapy between January 2014 and June 2018. We included patients with IPF who were treated with pirfenidone or nintedanib for ≥6 months. Pulmonary function tests (PFTs) were conducted after 6 months of initiation of treatment. We excluded patients with lung cancer, serious cardiovascular disease, serious infection, or collagen disease at the time of administration of pirfenidone or nintedanib. Also, we excluded patients using corticosteroids, immunosuppressants, or N-acetylcysteine during the observation period, because their therapies may affect the serum levels of the biomarkers.

### Pirfenidone and nintedanib treatment

The daily dose of pirfenidone was increased in a stepwise manner from 600 mg to 1200–1800 mg every 2 weeks, as previously described [15]. The median maximum dose was 1500 mg (inter-quartile range [IQR], 1200–1800 mg), and the median final dose was 1200 mg (IQR, 1200–1800 mg). Nintedanib was initiated at a daily dose of 300 mg. The dose was decreased depending on the severity of adverse events. The median final dose was 250 mg (IQR, 200–300 mg).

### Measurement of clinical parameters

All subjects were reviewed in terms of their clinical characteristics, PFT results (including FVC and DLco), arterial blood gas analysis, 6-min-walk test (6MWT), and serum SP-A, SP-D, and KL-6 levels at baseline (at the time of initiation of pirfenidone or nintedanib). The 6MWT was performed in accordance with the ATS guidelines [16]. Serum levels of SP-A, SP-D, and KL-6 were measured by enzyme-linked immunosorbent assays using commercial measurement kits as previously described [15]. We assessed the correlation between the parameters at baseline and those at 3 and 6 months, and the corresponding changes in FVC and DLco.

### Change in FVC and DLco, and definition of deterioration

Disease progression during pirfenidone and nintedanib therapy was evaluated based on PFTs at 6 months (including up to 9 months) from baseline [17]. “Progression” was defined as a ≥ 10% relative decline in FVC from baseline and/or ≥ 15% relative decline in DLco from baseline. Patients with progression were classified into the “progression group,” whereas the others were classified into the “stable group.”

### Changes in SP-A, SP-D, and KL-6

The relative changes in serum SP-A, SP-D, and KL-6 at 3 months and 6 months from baseline were evaluated.

### Statistical analysis

All data are expressed as median (IQR). The Mann–Whitney *U* test or Wilcoxon signed-rank test was used to compare the differences between the stable group and the progression group. To compare the levels of SP-A, SP-D, and KL-6 at baseline, 3, and 6 months in each group, we performed the Friedman and Wilcoxon signed-rank tests with Bonferroni correction, which are non-parametric tests for comparing multiple groups of the paired sample. Spearman’s rank correlation coefficient was used to evaluate the correlation between the changes in serum biomarker levels and the change in FVC and DLco. Logistic regression analysis was performedto identify factors that predicted disease stability at 6 months from administration of anti-fibrotic drugs. Factors associated with *p*-values < 0.20 in the univariate analysis were included in the multivariate analysis. Receiver-operating characteristic (ROC) curves were constructed for distinguishing between the stable and progression for changes in serum SP-A, SP-D, and KL-6. Cut-off levels were determined using the Youden index. The use of these cut-off levels allowed the calculation of the sensitivity and specificity.

All tests were performed at a significance level of *p* < 0.05 or Bonferroni corrected *p* < 0.05. Statistical analysis was performed using JMP 13.0 (SAS institute, Cary, NC, USA).

## Results

### Study population, patient characteristics, and clinical data

**Fig. 1:**
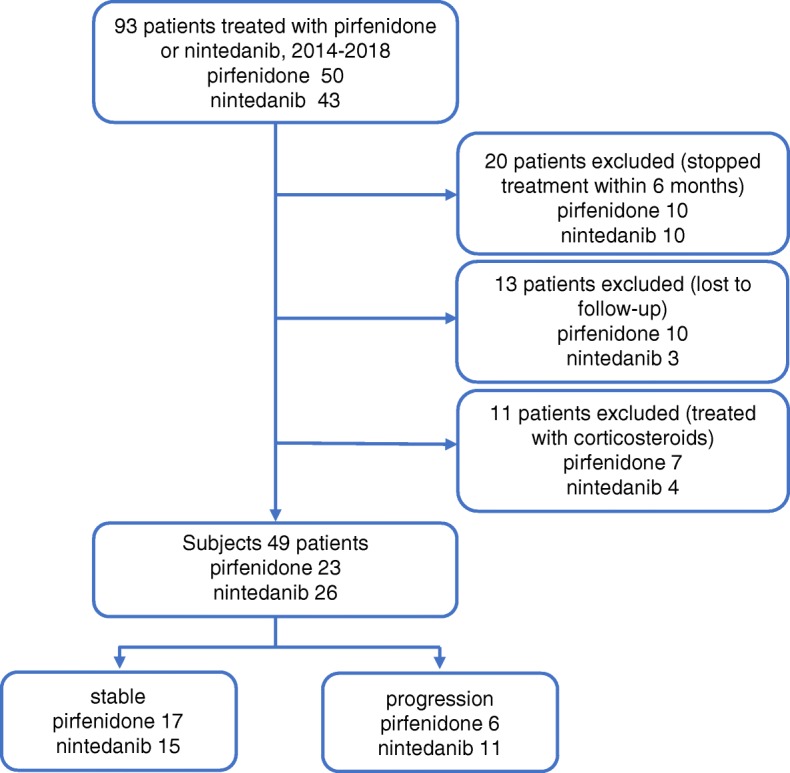
Schematic illustration of the study design and patient-selection criteria

Of the 93 patients with IPF who were initiated on pirfenidone or nintedanib therapy at our hospital, 49 patients were included in this analysis (pirfenidone, 23; nintedanib, 26) (Fig. 1). Twenty patients were excluded because the treatment was discontinued within 6 months. Reasons for discontinuation of pirfenidone treatment were disease progression (*n* = 3), nausea (*n* = 2), loss of appetite, liver function disorder, fever, xerophthalmia (*n* = 1, respectively). Reasons for discontinuation of nintedanib treatment were disease progression (n = 2), thrombocytopenia (n = 2), nausea (n = 2), diarrhea, liver function disorder, gastrointestinal bleeding, nasal bleeding, and rash (n = 1, respectively). Thirteen patients were lost to follow-up and were excluded. Eleven patients were excluded because they were treated with corticosteroids and immunosuppressant drugs during the observation period.

**Fig. 2:**
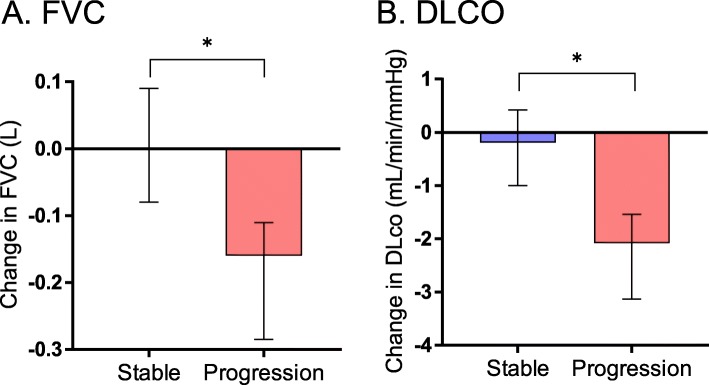
Rate of change in (**a**) FVC and (**b**) DLco in the initial 6 months. Changes in FVC and DLco in the stable group were significantly smaller than those in the progression group. Horizontal line indicates median concentration. The upper and lower limits of the line indicate the inter quartile range. Data were analyzed by Mann–Whitney *U* test. *p < 0.01 stable vs. progression group. FVC, forced vital capacity; DLco, diffusing capacity of the lung for carbon monoxide

Of the 49 patients included in this study, 32 patients were classified as the “stable group” (pirfenidone, 17; nintedanib, 15) and 17 patients were classified as the “progression group” (pirfenidone, 6; nintedanib, 11). The median observation period was 190.0 (169.8–263.2) days in the stable group and 183.0 (171.5–189.5) days in the progression group; there were no significant differences between the stable and the progression groups. Baseline characteristics were not significantly different between the stable and the progression groups (Table 1). Of the 26 patients treated with nintedanib, seven patients had past histories of treatment with pirfenidone (stable group, 4; progression group, 3). Two patients developed malignant tumor and were diagnosed with lung cancer. No patients developed acute exacerbations during the observation period. The changes in FVC and DLco from baseline to 6 months are shown in Fig. 2. The median change in FVC was 0.00 (− 0.08–0.09) L in the stable group and − 0.16 (− 0.29 to − 0.11) L in the progression group; the change in the stable group was significantly smaller than that in the progression group (*p* < 0.01). The median change in DLco was − 0.19 (− 1.00–0.42) mL/min/mmHg in the stable group and − 2.08 (− 3.13 to − 1.54) mL/min/mmHg in the progression group; the change in the stable group was significantly smaller than that in the progression group (p < 0.01).

**Table 1:** Baseline characteristics and clinical data

Variable	All subjects	Stable group	Progression group	*P*-value
	(*n* = 49)	(*n* = 32)	(*n* = 17)	
Sex M/F (n)	38/11	24/8	14/3	0.56
Age (yr)	69 (65–75)	70 (66–76)	68 (64–75)	0.38
Smokers/never-smokers (n)	36/13	23/9	13/4	0.73
Pack-years smoking	30 (0.8–51.5)	34 (5–52.1)	25 (0.8–49.5)	0.61
BMI	23.8 (22.1–25.8)	23.6 (22.1–25.5)	25.0 (21.7–26.1)	0.43
GAP stage (n), I/II/III	20/24/5	14/15/3	6/9/2	0.84
FVC (L)	2.42 (2.08–2.86)	2.40 (2.09–3.65)	2.64 (2.02–2.78)	0.83
% FVC (%)	76.6 (65.4–92.5)	77.4 (65.1–94.4)	76.6 (66.4–86.9)	0.78
DLco (mL/min/mmHg)	10.7 (9.22–13.5)	10.6 (9.25–13.5)	10.7 (8.81–14.3)	0.76
% DLco (%)	52.8 (44.3–59.8)	52.8 (44.4–57.6)	51.7 (43.6–70.3)	0.97
PaO_2_ at rest (Torr)	82.7 (78.2–88.6)	81.5 (77.2–91.6)	83.8 (78.4–87.3)	0.71
Minimum SpO_2_ during 6MWT (%)	91 (87.3–93)	91 (88–93)	90 (85.5–93)	0.42
6MWT Distance (meter)	410 (380–480)	420 (385–480)	400 (360–490)	0.94
SP-A (ng/mL)	57.8 (41.7–79.1)	60.3 (42.6–76.1)	56.6 (40.6–96.7)	0.83
SP-D (ng/mL)	243 (181–352)	241 (177–357)	246 (166–350)	0.97
KL-6 (U/mL)	901 (663–1494)	885 (595.3–1618)	901 (732–1336)	0.44
pirfenidone/nintedanib	23/26	17/15	6/11	0.23
Treatment history of anti-fibrotic drug (n), yes/no	7/42	4/28	3/14	0.66

Data are expressed as frequencies or medians (interquartile range). *P*-value: stable group vs. progression group.

*BMI* body mass index; *GAP* (gender [G], age [A], and 2 lung physiology variables [P] [FVC and DLco]); *FVC* forced vital capacity; *DLco* diffusing capacity of the lung for carbon monoxide; *PaO*_*2*_ partial pressure of arterial oxygen; *SpO*_*2*_ arterial oxygen saturation measured by pulse oximetry; *6MWT* 6 min-walk test; *SP* surfactant protein; *KL-6* Krebs von den Lungen-6

### Changes in serum biomarkers at 3 and 6 months

The levels of SP-A, SP-D, and KL-6 at baseline, 3, and 6 months are shown in Table 2. In the stable group, SP-A and KL-6 levels significantly decreased from baseline to 3 months and 6 months. On the other hand, in the progression group, SP-A showed a significant increase from baseline to 3 and 6 months. KL-6 tended to increase at 3 and 6 months; however, the difference was not significant. At 6 months, KL-6 level in the stable group was significantly lower than that in the progression group.

**Table 2:** Serum levels of SP-A, SP-D, and KL-6 at baseline, 3, and 6 months

	Baseline	3 months	6 months
Stable group			
SP-A (ng/mL)	60.3 (42.6–76.1)	53.1 (36.6–68.0) *	49.2 (37.1–71.7) *
SP-D (ng/mL)	241 (177–357)	194 (149–296)	195 (128–263)
KL-6 (U/mL)	885 (595–1618)	775 (581–1154) *	738 (503–1213) *^#^
Progression group			
SP-A (ng/mL)	56.6 (40.6–96.7)	67.0 (46.8–111.8) *	69.3 (42.7–117.8) *
SP-D (ng/mL)	246 (166–350)	261 (180–323)	224 (192–353)
KL-6 (U/mL)	901 (732–1337)	1195 (653–1821)	1237 (748–1570) ^#^

Data are expressed as medians (interquartile range).

*: Bonferroni-corrected *p* < 0.05 baseline vs. 3 or 6 months. ^#^: *p* < 0.05 stable group vs. progression group. *SP* surfactant protein; *KL-6* Krebs von den Lungen-6

**Fig. 3:**
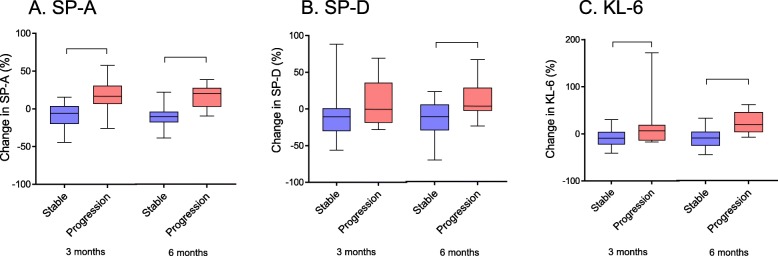
Relative change in (**a**) SP-A, (**b**) SP-D, and (**c**) KL-6 levels in the initial 3 and 6 months. Changes in serum SP-A at 3 and 6 months, SP-D at 6 months, and KL-6 at 3 and 6 months were significantly smaller in the stable group than the progression group (*p < 0.05). Data were analyzed by Mann–Whitney *U* test. Horizontal line indicates median concentration. The upper and lower limits of the box indicate the inter quartile range

The relative changes in serum biomarker levels from baseline to 3 and 6 months are shown in Fig. 3. The median change in SP-A at 3 and 6 months was − 6.0 (− 20.1–3.6) % and − 10.2 (− 17.9 to − 3.85) % in the stable group, and 16.7 (6.5–30.7) % and 20.2 (3.0–27.9) % in the progression group; the changes at 3 and 6 months in the stable group were significantly smaller than those in the progression group. The median change in SP-D at 3 and 6 months was − 10.6 (− 30.3–1.0) % and − 13.7 (− 32.1–2.2) % in the stable group and − 0.4 (− 18.6–35.8) % and 0.5 (− 6.0–24.7) % in the progression group; the changes at 6 months in the stable group were significantly smaller than those in the progression group. The median changes in KL-6 at 3 and 6 months were − 9.2 (− 22.5–4.1) % and − 15.0 (− 30.6−− 2.8) % in the stable group and 6.7 (− 14.0–18.9) % and 12.1 (− 3.6–36.6) % in the progression group; the changes at 3 and 6 months in the stable group were significantly smaller than those in the progression group.

### Pirfenidone and nintedanib subgroup evaluation

We performed subgroup analysis by disaggregating patients treated with pirfenidone and nintedanib. There was no significant difference between pirfenidone group and nintedanib group with respect to baseline characteristics (See Additional file 5: Table S1).

In the pirfenidone group, serum SP-A in the stable group was significantly lower than that in the progression group (*p* < 0.05); however, no significant difference was noted with respect to other baseline characteristics between the stable and progression subgroups of the pirfenidone and nintedanib groups. In both the pirfenidone and nintedanib groups, the changes in FVC and DLco from the baseline to 6 months in the stable group were significantly smaller than that in the progression group.

In patients treated with pirfenidone, the median change in SP-A at 3 and 6 months was − 6.0 (− 19.8–2.3) % and − 10.3 (− 20.1 to − 5.9) % in the stable group and 28.6 (− 3.8–41.1) % and 25.1 (0–33.9) % in the progression group; the changes at 3 and 6 months were significantly smaller in the stable group than in the progression group (*p* < 0.05) (See Additional file 1: Figure S1). The median change in SP-D at 6 months was − 12.7 (− 21.8–1.4) % in the stable group and 15.3 (− 5.8–26.7) % in the progression group; the change in the stable group was significantly smaller than that in the progression group (*p* < 0.05). The median change in KL-6 at 3 and 6 months was − 8.6 (− 16.9–7.4) % and − 8.5 (− 32.2–0.8) % in the stable group and 14.3 (− 0.5–86.1) % and 25.5 (7.5–44.3) % in the progression group; the changes at 3 and 6 months in the stable group were significantly smaller than those in the progression group (*p* < 0.05). In patients treated with nintedanib, the median change in SP-A at 3 and 6 months was − 5.0 (− 23.0–4.5) % and − 10.0 (− 18.5–5.6) % in the stable group and 14.7 (8.7–21.0) % and 18.7 (1.9–25.8) % in the progression group; the changes at 3 and 6 months in the stable group were significantly smaller than those in the progression group (*p* < 0.05). The median change in KL-6 at 6 months was − 16.6 (− 24.7 to − 4.7) % in the stable group and 6.8 (− 6.8–16.9) % in the progression group; the change was significantly smaller in the stable group than in the progression group (p < 0.05). The changes in SP-D were not significantly different between these groups.

### Correlation between serum biomarkers and PFTs

**Fig. 4:**
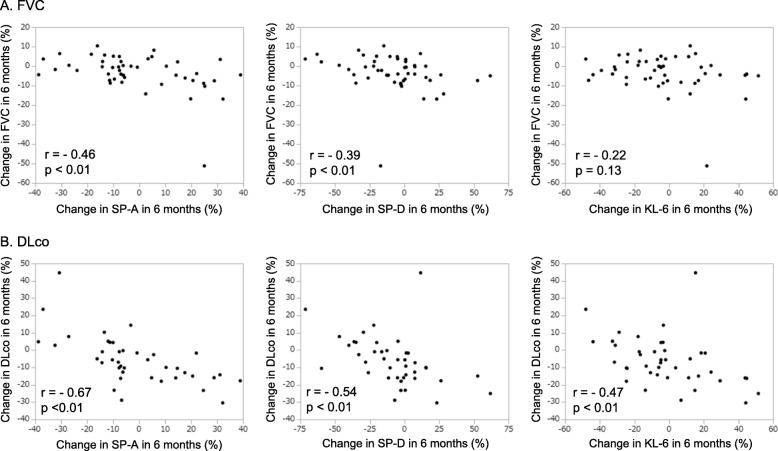
Correlation between changes in (**a**) FVC and (**b**) DLco and changes in SP-A, SP-D, and KL-6. (**a**) Decline in FVC showed a correlation with decrease in serum SP-A and SP-D (r = − 0.46 and r = − 0.39, respectively, p < 0.01). (**b**) Decline in DLco showed a correlation with decrease in serum SP-A, SP-D, and KL-6 (r = − 0.67, r = − 0.54, and r = − 0.47 respectively, p < 0.01). Spearman’s correlation coefficients were used to analyze the linear relationship between the variables

The correlation between the changes in serum biomarker levels and the change in FVC and DLco are shown in Fig. 4. We observed a negative correlation between the change in FVC at 6 months and the changes in SP-A (correlation coefficient: − 0.46, *p* < 0.01) and SP-D (correlation coefficient: − 0.39, p < 0.01) at 6 months. We also observed a negative correlation between the change in DLco and the changes in SP-A (correlation coefficient: − 0.67, p < 0.01), SP-D (correlation coefficient: − 0.54, p < 0.01) and KL-6 (correlation coefficient: − 0.47, p < 0.01).

In patients treated with pirfenidone, we observed a negative correlation between the change in FVC at 6 months and change in SP-A (correlation coefficient: − 0.60, *p* < 0.01) and SP-D (correlation coefficient: − 0.49, *p* < 0.05) at 6 months; similarly, we observed a negative correlation between the change in DLco and changes in SP-A (correlation coefficient: − 0.59, *p* < 0.05), SP-D (correlation coefficient: − 0.56, p < 0.05), and KL-6 (correlation coefficient: − 0.48, p < 0.05) **(**See Additional file 6: Table S2). In patients treated with nintedanib, we observed a negative correlation between the change in DLco and changes in SP-A (correlation coefficient: − 0.61, *p* < 0.01), SP-D (correlation coefficient: − 0.42, p < 0.05), and KL-6 (correlation coefficient: − 0.47, p < 0.05).

### Predictive factors of therapeutic effect of anti-fibrotic drugs

We performed logistic regression analysis to identify factors that predicted therapeutic outcome at 6 months (Table 3). On univariate analysis, the change in SP-A at 3 months (odd’s ratio [OR] 0.89, 95% CI 0.82–0.96, *p* < 0.01), KL-6 at 3 months (OR 0.96, 95% CI 0.92–0.99, p < 0.01), SP-A in 6 months (OR 0.89, 95% CI 0.84–0.95, p < 0.01), SP-D in 6 months (OR 0.95, 95% CI 0.92–0.99, *p* < 0.01), and KL-6 in 6 months (OR 0.93, 95% CI 0.89–0.97, p < 0.01) showed a significant association with outcomes. Three multivariate analysis models were constructed (Table 4). When the change in SP-A, SP-D, and KL-6 at 3 months were entered in the 3 months model (model 1), the change in SP-A at 3 months was a significant predictive factor (OR 0.88, 95% CI 0.80–0.97, *p* < 0.01). When the change in SP-A, SP-D, and KL-6 at 6 months were entered in the 6 months model (model 2), the change in SP-A at 6 months was a significant predictive factor (OR 0.90, 95% CI 0.84–0.97, p < 0.01). In the 3 months model and 6 months model (model 3), the changes in SP-A at 3 and 6 months were significant predictive factors (OR 0.89, 95% CI 0.76–0.98, *p* < 0.05; OR 0.88, 95% CI 0.76–0.96, p < 0.01; respectively).

**Table 3:** Prediction of stability at 6 months from administration of anti-fibrotic drugs in univariate analysis

Variable	OR (95% CI)	*P*-value
Sex, Male	0.64 (0.15–2.83)	0.55
Age	1.05 (0.96–1.14)	0.24
Smokers	1.27 (0.33–4.96)	0.72
Pack-years smoking	1.01 (0.99–1.03)	0.59
BMI	0.89 (0.74–1.08)	0.22
% FVC (%)	1.01 (0.98–1.04)	0.61
% DLco (%)	1.00 (0.95–1.04)	0.83
PaO_2_ at rest (Torr)	1.01 (0.94–1.09)	0.71
Minimum SpO_2_ during 6MWT (%)	1.06 (0.93–1.21)	0.37
6MWT Distance (m)	1.00 (0.99–1.01)	0.80
SP-A (ng/mL)	0.99 (0.98–1.01)	0.30
SP-D (ng/mL)	1.00 (1.00–1.00)	0.64
KL-6 (U/mL)	1.00 (1.00–1.00)	0.65
Pirfenidone	2.01 (0.62–6.99)	0.23
Treatment history of anti-fibrotic drugs (n)	0.69 (0.14–3.53)	0.66
Change in SP-A in 3 months (%)	0.89 (0.82–0.96)	< 0.01
Change in SP-D in 3 months (%)	0.98 (0.96–1.00)	0.12
Change in KL-6 in 3 months (%)	0.96 (0.92–0.99)	< 0.01
Change in SP-A in 6 months (%)	0.89 (0.84–0.95)	< 0.01
Change in SP-D in 6 months (%)	0.95 (0.92–0.99)	< 0.01
Change in KL-6 in 6 months (%)	0.93 (0.89–0.97)	< 0.01

*OR* odd’s ratio; *BMI* body mass index; *FVC* forced vital capacity; *DLco* diffusing capacity of the lung for carbon monoxide; *PaO*_*2*_ partial pressure of arterial oxygen; *SpO*_*2*_ arterial oxygen saturation measured by pulse oximetry; *6MWT* 6 min-walk test; *SP* surfactant protein; *KL-6* Krebs von den Lungen-6

**Table 4:** Prediction of stability at 6 months from administration of anti-fibrotic drugs in multivariate analysis

variable	model 1		model 2		model 3 (model 1 + model 2)	
	OR (95% CI)	*P*-value	OR (95% CI)	*P*-value	OR (95% CI)	*P*-value
Change in SP-A in 3 months (%)	0.88 (0.80–0.97)	< 0.01	–	–	0.89 (0.76–0.98)	< 0.05
Change in SP-D in 3 months (%)	1.10 (0.98–1.05)	0.28	–	–	1.04 (0.99–1.13)	0.09
Change in KL-6 in 3 months (%)	0.99 (0.95–1.03)	0.67	–	–	0.99 (0.92–1.04)	0.71
Change in SP-A in 6 months (%)	–	–	0.90 (0.84–0.97)	< 0.01	0.88 (0.76–0.96)	< 0.01
Change in SP-D in 6 months (%)	–	–	0.98 (0.92–1.03)	0.37	0.98 (0.90–1.04)	0.49
Change in KL-6 in 6 months (%)	–	–	0.95 (0.90–1.01)	0.05	0.96 (0.87–1.03)	0.30

*OR* odd’s ratio; *SP* surfactant protein; *KL-6* Krebs von den Lungen-6

We analyzed the sensitivity and specificity to distinguish between the stable and the progressive patients for changes in SP-A, SP-D, and KL-6. The sensitivity and specificity estimated according to the ROC curve analyses are shown in Table S3 (See Additional file 7). The sensitivity, specificity and the AUC of change in SP-A in 3 months were 93, 75%, and 0.89, respectively. The sensitivity, specificity and AUC of change in SP-A in 6 months were 81, 81%, and 0.89, respectively.

## Discussion

We report an association between changes in serum SP-A, SP-D, and KL-6 levels and change in FVC and DLco of patients with IPF treated with anti-fibrotic drugs. Patients with IPF who maintained their FVC and DLco showed a significant decrease in SP-A and KL-6 at 3 and 6 months. On the other hand, those who showed decline in FVC and DLco had increased SP-A levels at 3 and 6 months. The relative changes in serum biomarkers were significantly smaller in the stable group than in the progression group. The changes in serum biomarker levels showed a significant correlation with changes in FVC and DLco. In particular, changes in SP-A levels most closely reflected the outcomes of anti-fibrotic therapy. This study indicates that SP-A may be used as a biomarker to predict the outcomes of anti-fibrotic drug therapy.

Serum levels of SP-A, SP-D, and KL-6 have been shown to be useful in predicting prognosis and monitoring disease activity in patients with IPF [12, 18, 19]. In previous studies, SP-A was found to be a predictor of early mortality in patients with IPF [19, 20]. In our previous studies, patients with high levels of SP-D tended to exhibit worsening restrictive impairment and poor prognosis [13, 21]. Serum KL-6 levels were shown to correlate with the severity and prognosis of patients with IPF [14, 22]; in addition, serial change in KL-6 levels was a prognostic factor for patients with IPF [23, 24]. However, the usefulness of these serum biomarkers in patients undergoing anti-fibrotic drug therapy is not well characterized. A few studies have investigated the clinical relevance of SP-D and KL-6 levels in the context of pirfenidone therapy. For example, SP-D was shown to be prognostic marker in patients with IPF treated with pirfenidone [15]. Similarly, in a study by Okuda, et al., levels of KL-6 and SP-D were decreased by pirfenidone therapy [25]. The present study showed that changes in serum biomarkers may serve as useful markers for clinical evaluation and monitoring of therapeutic efficacy in patients with IPF treated with anti-fibrotic drugs. Thus, our study provides novel insights for further investigations to verify the clinical relevance of these serum biomarkers in the context of anti-fibrotic therapy for IPF.

In the present study, serum biomarker levels at baseline did not show any significant differences between the stable group and the progression group. In our previous study, baseline level of SP-D was found to predict mortality of patients with IPF treated with pirfenidone [15]. This discrepancy may be partly explained by the shorter duration of follow-up and the difference in the definition of deterioration (including not only FVC but also DLCO) in the present study. In addition, previous study lacked longitudinal assessment to determine whether changes in serum biomarker levels reflect the therapeutic effect. In a study by Sokai, et al., serial changes in KL-6 in patients with IPF showed a significant correlation with changes in FVC and DLco; in addition, serial change in KL-6 level was a predictor of mortality; however, most of patients were not treated with anti-fibrotic drugs [24]. Our study is the first to demonstrate that serial changes in SP-A and KL-6 reflect the decline in pulmonary function of patients with IPF treated with anti-fibrotic drugs. These results suggest that serial measurement of serum biomarker levels is more useful for monitoring patients with IPF and their response to anti-fibrotic therapy.

IPF is a progressive lung disease characterized by decline in FVC. In both ASCEND and INPULSIS-1/2 phase III trials, the primary endpoint was the annual rate of decline in FVC [4, 5]. Currently, alleviation of the rate of decline in FVC is the only criterion to ascertain the therapeutic efficacy of pirfenidone and nintedanib. No other biomarker of the therapeutic effect of anti-fibrotic therapy has been elucidated. It is difficult to evaluate the degree of efficacy of pirfenidone and nintedanib by pulmonary function tests alone. Furthermore, it is difficult to perform pulmonary function tests frequently in patients with severe IPF owing to the following reasons: 1) it imposes a physical burden on patients with serious disease; 2) the results depend on the patient’s efforts; 3) maximal effort may be difficult because of cough; 4) DLco cannot be measured in severe cases. Therefore, use of pulmonary function tests in conjunction with serum biomarkers will help reduce the burden on patients and allow for more frequent assessments. Moreover, this will facilitate more accurate assessment of the therapeutic outcomes of pirfenidone and nintedanib.

In IPF, serial changes in FVC and DLco at 6 or 12 months show greater prognostic value than baseline data [26, 27]. Even a decline in FVC by 5–10% at 6 months was associated with increased mortality [27]. In the study by Du Bois et al., change in percent-predicted FVC over 6 months was a strong predictor of death over 1-year period [28]. Risk of death was nearly five-fold higher for patients with > 10% decline in percent-predicted FVC, and two-fold higher for those with 5–10% decline [28]. The rate of decline in FVC at 6 months is associated with the subsequent prognosis. Although the evaluation period in this study was 6 months, it was considered to be an appropriate period for prognostic assessment. Furthermore, absence of signs of therapeutic effect at 6 months should prompt reconsideration of treatment such as a change to another anti-fibrotic drug.

Patients with IPF show increased production of SP-A, SP-D, and KL-6 by alveolar type 2 epithelial cells in the lung tissues [11, 12, 29]. Levels of SP-A and SP-D in bronchoalveolar lavage (BAL) fluid in patients with IPF were shown to decrease as compared to healthy subjects, whereas levels of KL-6 were shown to increase [29–31]. It is considered that serum SP-A, SP-D, and KL-6 levels are elevated due to increased vascular leakage from the alveolar space, probably due to epithelial detachment, basement membrane injury, and capillary fragility [15, 29]. In this study, serum SP-A and KL-6 levels were found to decrease after treatment with anti-fibrotic drugs. These results may be explained by the following mechanisms of action of nintedanib: 1) suppression of vascular permeability and capillary angiogenesis via VEGF; 2) repair of lung tissue with reduction of fibroblast migration and cell proliferation with PDGF and FGF; 3) suppression of the production of SP-A in alveolar type 2 epithelial cells by FGF. Pirfenidone suppresses the production of TGF-β and inflammatory cytokines in addition to PDGF and FGF [32, 33]. It may also reduce the leakage by suppressing the production of SP-A and KL-6 by epithelial cells and by reducing abnormal tissue repair.

In the present study, in both pirfenidone and nintedanib groups, there was no difference in the reduction rate of pulmonary function between the stable group and the progression group; in addition, the number of patients in the stable group was also similar. Although the difference between the therapeutic effect of pirfenidone and nintedanib has not been clarified, the therapeutic effects may be comparable [34]. Moreover, it is not clear whether change from one anti-fibrotic drug to the other accrues any additional benefit. In the present study population, seven patients with IPF were shifted from pirfenidone to nintedanib; however, there was no difference between the stable and progression groups with respect to history of treatment with pirfenidone. Our results also showed additional therapeutic benefit in these patients, although the number of patients was small. A previous report also documented the therapeutic benefit of change in anti-fibrotic drug in a small number of cases; this phenomenon may be attributable to the different mechanisms of action of these drugs [35]. Further studies with larger sample are required to obtain more definitive evidence of the benefit of treatment with two anti-fibrotic drugs, and to determine the optimal sequence of use of anti-fibrotic drugs.

The present study has some limitations. First, this was a single-center retrospective study with a small sample size. Further, the number of subjects has decreased because 11 patients, including 3 cases where patients developed acute exacerbation before anti-fibrotic drug administration, were excluded because corticosteroid therapy was considered to affect serum biomarker expression. In the present study, we evaluated 60 people, including 11 patients who received treatment with corticosteroids and confirmed that the results were the same (see Additional files 2,3,4,8,9,10,11: Figure S2–S4, Table S4–S7). Therefore, treatment with steroids does not affect the results, and we thus believe that sampling errors will not occur in this study that has continued for more than 6 months. Second, nintedanib has been available in Japan since August 2015; therefore, only pirfenidone was available for use during the initial period of the study reference period. The nintedanib group included some patients who had a history of treatment with pirfenidone. However, there were no significant differences between the pirfenidone and nintedanib groups with respect to baseline characteristics, clinical data, and outcomes. Third, this study did not include a group that was not treated with anti-fibrotic drugs; thus, it may not be concluded that the amelioration of decline in FVC and DLco was attributable to anti-fibrotic drug therapy. However, it is difficult to plan comparative studies on the treatment of IPF in an era when anti-fibrotic drugs have become the standard therapy for treating IPF. Finally, the observation period was 6 months, and long-term efficacy and prognosis have not been investigated. It is necessary to clarify the relationship of serum biomarkers with therapeutic effect and prognosis over a longer term.

## Conclusion

Changes in serum SP-A levels reflected the outcomes of anti-fibrotic drug therapy. Serial measurements of SP-A may serve as a useful therapeutic biomarker and may help guide the management of IPF.

## Supplementary information

**Additional file 1: Figure S1.** Relative change in SP-A, SP-D, and KL-6 in patients treated with (A) pirfenidone and (B) nintedanib. (A) Change in SP-A at 3 and 6 months, SP-D at 6 months, and KL-6 at 3 and 6 months were significantly smaller in the stable group than the progression group (*p* < 0.05). (B) Change in SP-A at 3 and 6 months and KL-6 at 6 months were significantly smaller in the stable group than the progression group (p < 0.05)

**Additional file 2: Figure S2.** Rate of change in (A) FVC and (B) DLco in the initial 6 months of population which included patients who used corticosteroids. Changes in FVC and DLco in the stable group were significantly smaller than those in the progression group (*p* < 0.01)

**Additional file 3: Figure S3.** Relative change in (A) SP-A, (B) SP-D, and (C) KL-6 levels in the initial 3 and 6 months of population which included patients who used corticosteroids. Changes in SP-A, SP-D, and KL-6 at 3 and 6 months were significantly smaller in the stable group than the progression group (p < 0.05)

**Additional file 4: Figure S4.** Correlation between changes in (A) FVC and (B) DLco and changes in SP-A, SP-D, and KL-6 of population which included patients who used corticosteroids. (A) Change in FVC showed a negative correlation with changes in SP-A and SP-D (p < 0.01). (B) Change in DLco showed a negative correlation with changes in SP-A, SP-D, and KL-6 (p < 0.01)

**Additional file 5: Table S1.** Baseline characteristics and clinical data of the pirfenidone and nintedanib groups

**Additional file 6: Table S2.** Correlation between change in SP-A, SP-D, and KL-6 and change in pulmonary function in 6 months of the pirfenidone and nintedanib groups

**Additional file 7: Table S3.** Sensitivity and specificity for distinguish between the stable and the progressive patients for changes in SP-A, SP-D, and KL-6

**Additional file 8: Table S4.** Baseline characteristics and clinical data of population which included patients who used corticosteroids

**Additional file 9: Table S5.** Serum levels of SP-A, SP-D, and KL-6 at baseline, 3, and 6 months of population which included patients who used corticosteroids

**Additional file 10: Table S6.** Prediction of stability at 6 months from administration of anti-fibrotic drugs in univariate analysis of population which included patients who used corticosteroids

**Additional file 11: Table S7.** Prediction of stability at 6 months from administration of anti-fibrotic drugs in multivariate analysis of population which included patients who used corticosteroids

## Data Availability

All datasets are available from the corresponding author on reasonable request.

## Abbreviations

6MWT: 6-min walk test
ALAT: Latin American Thoracic Association
ATS: American Thoracic Society
AUC: Area under the curve
BAL: Bronchoalveolar lavage
DLco: Diffusing capacity of the lung for carbon monoxide
ERS: European Respiratory Society
FGFs: Fibroblast growth factors
FVC: Forced vital capacity
HRCT: High-resolution computed tomography
IPF: Idiopathic pulmonary fibrosis
IQR: Interquartile range
JRS: Japanese Respiratory Society
KL-6: Krebs von den Lungen-6
OR: Odds ratio
PaO_2_: Partial pressure of oxygen in arterial blood
PDGFs: Platelet-derived growth factors
PFT: Pulmonary function test
ROC: Receiver operating characteristic
SP-A: Surfactant protein-A
SP-D: Surfactant protein-D
SpO_2_: Peripheral capillary oxygen saturation
TGF-β: Transforming growth factor-beta
VEGF: Vascular endothelial growth factor
