# Correction to: Surfactant protein A as a biomarker of outcomes of anti-fibrotic drug therapy in patients with idiopathic pulmonary fibrosis

**DOI:** 10.1186/s12890-020-1118-x

**Published:** 2020-05-07

**Authors:** Takumi Yoshikawa, Mitsuo Otsuka, Hirofumi Chiba, Kimiyuki Ikeda, Yuki Mori, Yasuaki Umeda, Hirotaka Nishikiori, Koji Kuronuma, Hiroki Takahashi

**Affiliations:** Department of Respiratory Medicine and Allergology, Sapporo Medical University School of Medicine, 1-37, South 1-West 16, Chuo-ku, Sapporo, Hokkaido 060-8543 Japan

**Correction to: BMC Pulm Med (2020) 20:27**

**https://doi.org/10.1186/s12890-020-1060-y**

Following publication of the original article [1], the authors have flagged that there is an error in Fig. 3.

Namely, asterisks indicating significant difference are missing in the figure.

Please see the correct version of Fig. 3 in this correction article.

The authors apologize for any inconvenience caused.

**Fig. 3:**
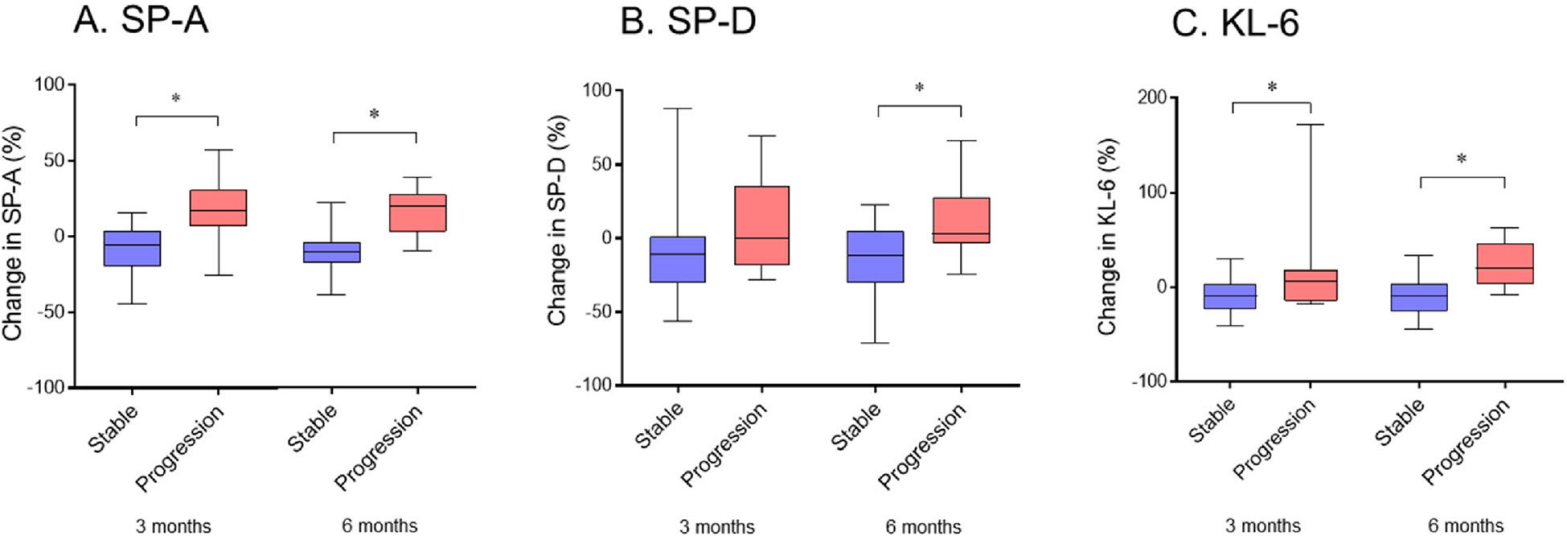
Relative change in SP-A, SP-D, and KL-6 levels in the initial 3 and 6 months in the stable and progression groups. Changes in SP-A at 3 and 6 months, SP-D at 6 months, and KL-6 at 3 and 6 months were significantly smaller in the stable group than the progression group (*p < 0.05). Data were analyzed by Mann-Whitney U test. Horizontal line indicates median concentration. The upper and lower limits of the box indicate the inter-quartile range
