# Supplementary material for: Surfactant protein A as a biomarker of outcomes of anti-fibrotic drug therapy in patients with idiopathic pulmonary fibrosis
Source: BMC Pulm Med. 2020 Jan 31;20:27. doi: 10.1186/s12890-020-1060-y (PMC6995128; doi:10.1186/s12890-020-1060-y)
Supplement: Supplementary file 1 — Additional file 1: Figure S1. Relative change in SP-A, SP-D, and KL-6 in patients treated with (A) pirfenidone and (B) nintedanib. (A) Change in SP-A at 3 and 6 months, SP-D at 6 months, and KL-6 at 3 and 6 months were significantly smaller in the stable group than the progression group (p < 0.05). (B) Change in SP-A at 3 and 6 months and KL-6 at 6 months were significantly smaller in the stable group than the progression group (p < 0.05) [file 12890_2020_1060_MOESM1_ESM.docx]

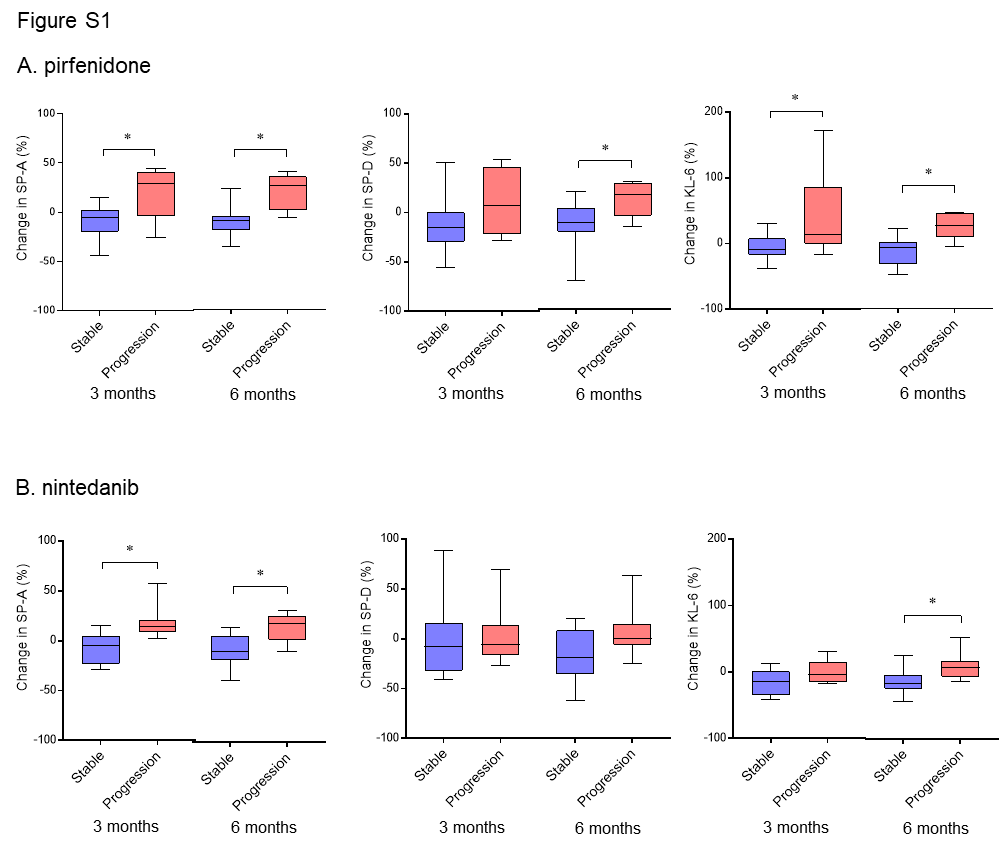


**Figure S1. Relative change in SP-A, SP-D, and KL-6 in patients treated with (A) pirfenidone and (B) nintedanib.**
