# Supplementary material for: Surfactant protein A as a biomarker of outcomes of anti-fibrotic drug therapy in patients with idiopathic pulmonary fibrosis
Source: BMC Pulm Med. 2020 Jan 31;20:27. doi: 10.1186/s12890-020-1060-y (PMC6995128; doi:10.1186/s12890-020-1060-y)
Supplement: Supplementary file 2 — Additional file 2: Figure S2. Rate of change in (A) FVC and (B) DLco in the initial 6 months of population which included patients who used corticosteroids. Changes in FVC and DLco in the stable group were significantly smaller than those in the progression group (p < 0.01) [file 12890_2020_1060_MOESM2_ESM.docx]

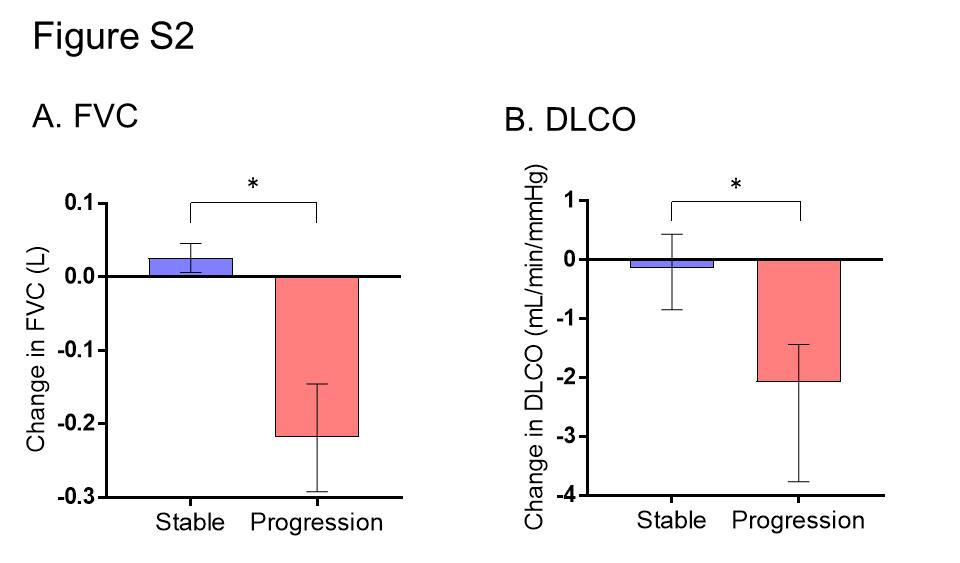


**Figure S2. Rate of change in (A) FVC and (B) DLco in the initial 6 months of population which included patients who used corticosteroids.**
