# Supplementary material for: Surfactant protein A as a biomarker of outcomes of anti-fibrotic drug therapy in patients with idiopathic pulmonary fibrosis
Source: BMC Pulm Med. 2020 Jan 31;20:27. doi: 10.1186/s12890-020-1060-y (PMC6995128; doi:10.1186/s12890-020-1060-y)
Supplement: Supplementary file 3 — Additional file 3: Figure S3. Relative change in (A) SP-A, (B) SP-D, and (C) KL-6 levels in the initial 3 and 6 months of population which included patients who used corticosteroids. Changes in SP-A, SP-D, and KL-6 at 3 and 6 months were significantly smaller in the stable group than the progression group (p < 0.05) [file 12890_2020_1060_MOESM3_ESM.docx]

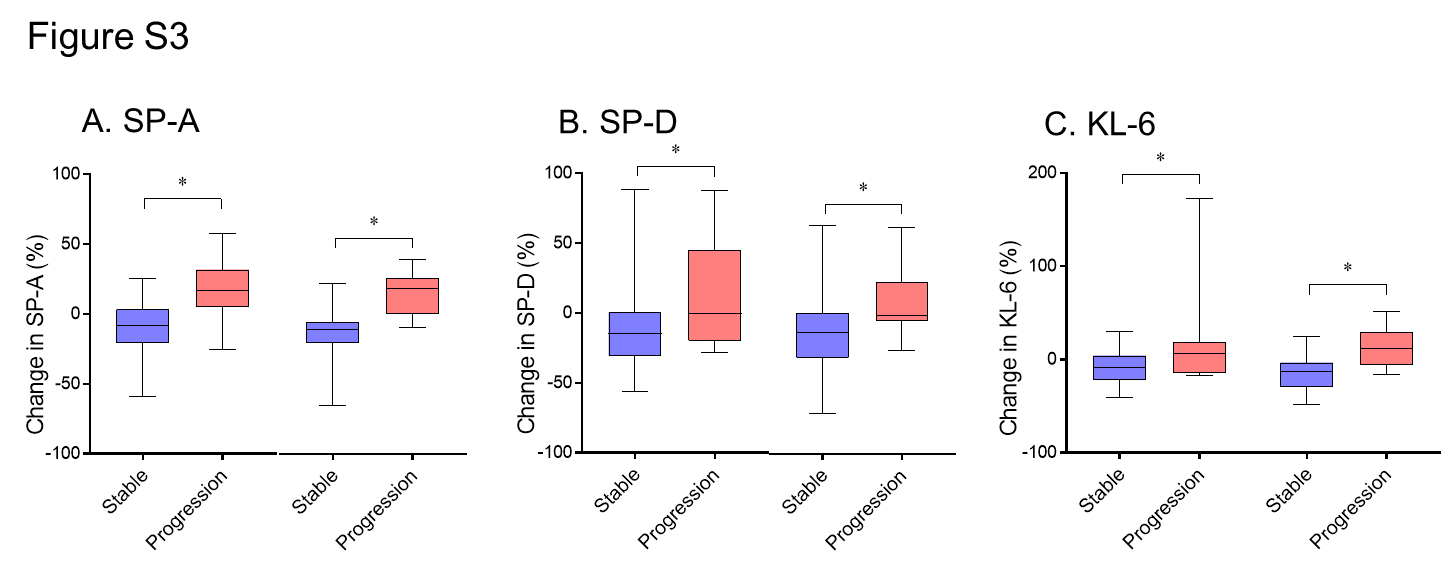


**Figure S3. Relative change in (A) SP-A, (B) SP-D, and (C) KL-6 levels in the initial 3 and 6 months** **of population which included patients who used corticosteroids.**
