# Supplementary material for: Surfactant protein A as a biomarker of outcomes of anti-fibrotic drug therapy in patients with idiopathic pulmonary fibrosis
Source: BMC Pulm Med. 2020 Jan 31;20:27. doi: 10.1186/s12890-020-1060-y (PMC6995128; doi:10.1186/s12890-020-1060-y)
Supplement: Supplementary file 4 — Additional file 4: Figure S4. Correlation between changes in (A) FVC and (B) DLco and changes in SP-A, SP-D, and KL-6 of population which included patients who used corticosteroids. (A) Change in FVC showed a negative correlation with changes in SP-A and SP-D (p < 0.01). (B) Change in DLco showed a negative correlation with changes in SP-A, SP-D, and KL-6 (p < 0.01) [file 12890_2020_1060_MOESM4_ESM.docx]

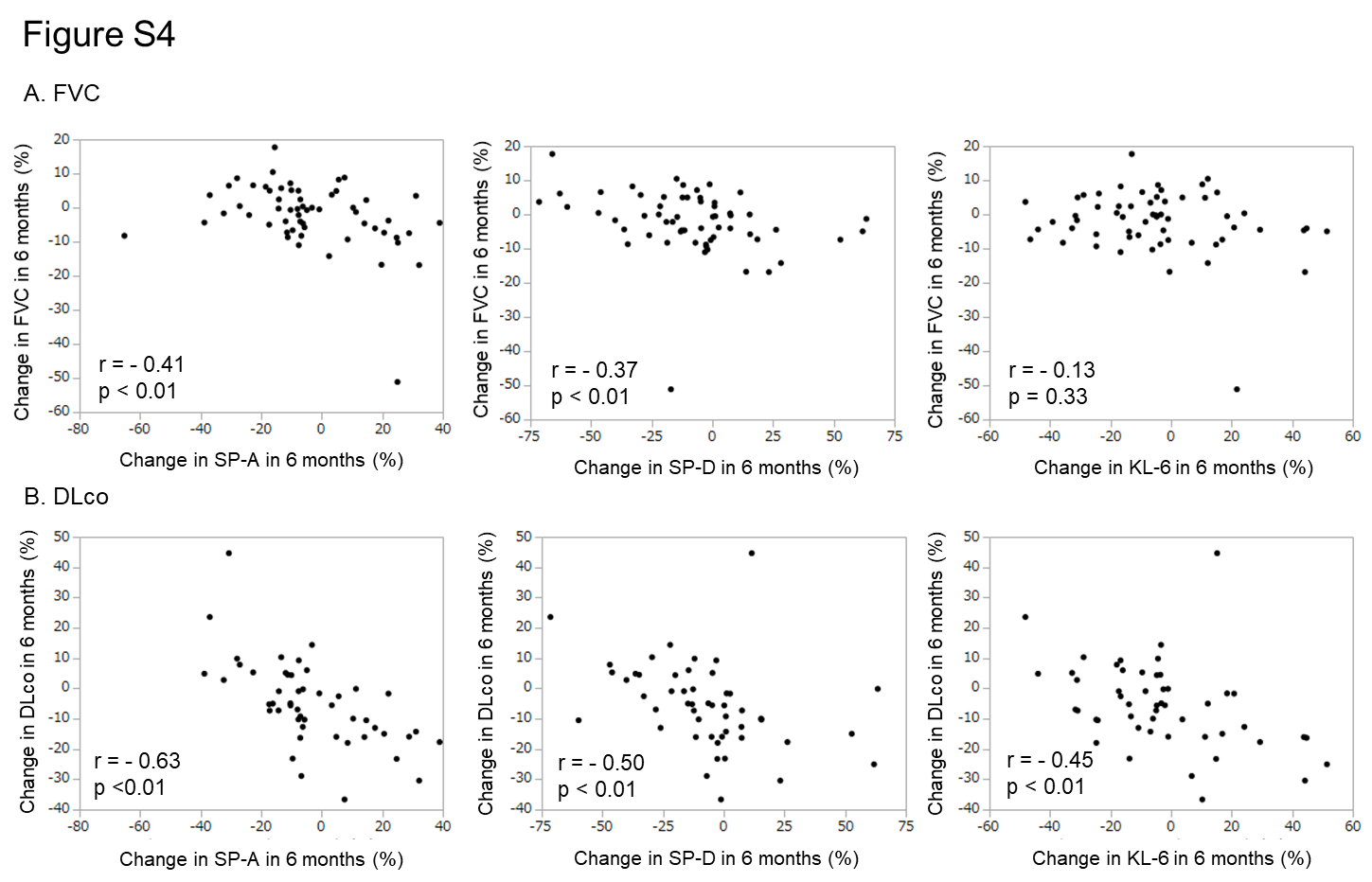


**Figure S4.** **Correlation between changes in (A) FVC and (B) DLco and changes in SP-A, SP-D, and KL-6 of population which included patients who used corticosteroids.**
