# Supplementary material for: Surfactant protein A as a biomarker of outcomes of anti-fibrotic drug therapy in patients with idiopathic pulmonary fibrosis
Source: BMC Pulm Med. 2020 Jan 31;20:27. doi: 10.1186/s12890-020-1060-y (PMC6995128; doi:10.1186/s12890-020-1060-y)
Supplement: Supplementary file 5 — Additional file 5: Table S1. Baseline characteristics and clinical data of the pirfenidone and nintedanib groups [file 12890_2020_1060_MOESM5_ESM.docx]

| **Table S1. Baseline characteristics and clinical data of the pirfenidone and nintedanib groups.** | | | | | |  |
| --- | --- | --- | --- | --- | --- | --- |
|  | pirfenidone group | | | nintedanib group | | |
|  | (n = 23) | | | (n = 26) | | |
|  | stable | progression | *P*-value | stable | progression | *P-*value |
| **Variable** | (n = 17) | (n = 6) |  | (n = 15) | (n = 11) |  |
| **Sex M/F (n)** | 14/3 | 5/1 | 0.96 | 10/5 | 9/2 | 0.39 |
| **Age (yr)** | 69 (66–75) | 67 (58–70) | 0.21 | 70 (66–76) | 70 (64–75) | 0.79 |
| **Smoker /never-smokers (n)** | 14/3 | 5/1 | 0.96 | 9/6 | 8/3 | 0.50 |
| **Pack-years smoking** | 30 (21–48) | 23.8 (1.1–52.8) | 0.83 | 38 (0–60) | 25 (0–52) | 0.58 |
| **BMI** | 23.4 (22.1–26.0) | 22.9 (20.2–26.1) | 0.55 | 24.2 (19.8–25.0) | 25.0 (22.8–26.3) | 0.13 |
| **GAP stage (n), I/II/III** | 9/6/2 | 1/4/1 | 0.30 | 5/9/1 | 5/5/1 | 0.76 |
| **FVC (L)** | 2.42 (2.17–2.97) | 2.68 (1.99–2.75) | 0.55 | 2.19 (1.96–2.75) | 2.64 (2.03–3.03) | 0.39 |
| **%FVC (%)** | 79.0 (70.2–106.1) | 71.2 (62.5–83.9) | 0.27 | 70.2 (62.8–89.3) | 76.8 (71.7–108.1) | 0.64 |
| **ΔFVC (L)** | −0.02 (−0.09–0.10) | −0.28 (−0.68–−0.11) | <0.01 | 0.01 (−0.04–0.09) | −0.16 (−0.25–−0.11) | <0.05 |
| **DLco (mL/min/mm Hg)** | 12.1 (10.6–14.2) | 10.2 (9.4–11.4) | 0.14 | 10.0 (8.8–10.6) | 12.6 (8.4–15.6) | 0.23 |
| **%DLco (%)** | 55.3 (49.2–69.3) | 51.7 (37.9–56.9) | 0.29 | 48.2 (41.9–55.6) | 53.3 (41.8–72.8) | 0.43 |
| **ΔDLco (mL/min/mm Hg)** | −0.13 (−0.85–0.79) | −1.81 (−3.77–−1.64) | <0.05 | −0.27 (−1.26–0.42) | −2.19 (−2.82–−1.43) | <0.01 |
| **PaO_2_ at rest (Torr)** | 85 (79–93) | 84 (79–88) | 0.48 | 80 (75–87) | 84 (78–87) | 0.72 |
| **Minimum SpO_2_ during 6MWT (%)** | 92 (89.5–95) | 89 (83–94.3) | 0.22 | 91 (85.3–92.3) | 91 (86–93) | 0.68 |
| **6MWT Distance (m)** | 420 (365–490) | 440 (365–485) | 0.92 | 400 (389–480) | 400 (360–530) | 0.85 |
| **SP-A (ng/mL)** | 52.6 (37.1–73.3) | 91.4 (66.2–194) | <0.05 | 64.5 (49.3–79.1) | 48.1 (37.6–63.7) | 0.12 |
| **SP-D (ng/mL)** | 217 (122–404) | 267 (232–427) | 0.28 | 247 (198–342) | 222 (139–302) | 0.23 |
| **KL-6 (U/mL)** | 922 (542–2130) | 1103 (892–1388) | 0.42 | 862 (595–1574) | 860 (673–1413) | 0.88 |
| **Treatment history of anti-fibrotic drug (n), yes/no** | 0/17 | 0/6 | – | 4/11 | 3/8 | 0.94 |
| Data are expressed as frequencies or medians (interquartile range). *P* < 0.05: stable group vs. progression group. BMI = body mass index; GAP = (gender [G], age [A], and 2 lung physiology variables [P] [FVC and DLco]); FVC = forced vital capacity; DLco = diffusing capacity of the lung for carbon monoxide; ΔFVC = change in FVC from baseline to 6 months; ΔDLco = change in DLco from baseline to 6 months; PaO_2_ = partial pressure of arterial oxygen; SpO_2_ = arterial oxygen saturation measured by pulse oximetry; 6MWT = 6 minute-walk test; SP = surfactant protein; KL-6 = Krebs von den Lungen-6 | | | | | |  |
