# Supplementary material for: Surfactant protein A as a biomarker of outcomes of anti-fibrotic drug therapy in patients with idiopathic pulmonary fibrosis
Source: BMC Pulm Med. 2020 Jan 31;20:27. doi: 10.1186/s12890-020-1060-y (PMC6995128; doi:10.1186/s12890-020-1060-y)
Supplement: Supplementary file 6 — Additional file 6: Table S2. Correlation between change in SP-A, SP-D, and KL-6 and change in pulmonary function in 6 months of the pirfenidone and nintedanib groups [file 12890_2020_1060_MOESM6_ESM.docx]

| **Table S2. Correlation between change in SP-A, SP-D, and KL-6 and change in pulmonary function in 6 months of the pirfenidone and nintedanib groups.** | | | |
| --- | --- | --- | --- |
| **correlation** | **relative change in biomarker** | ***r*** | ***P*-value** |
| **Pirfenidone** |  |  |  |
| relative change in FVC | SP-A | −0.60 | <0.01 |
|  | SP-D | −0.49 | <0.05 |
|  | KL-6 | −0.16 | 0.47 |
| relative change in DLco | SP-A | −0.59 | <0.05 |
|  | SP-D | −0.56 | <0.05 |
|  | KL-6 | −0.48 | <0.05 |
| **Nintedanib** |  |  |  |
| relative change in FVC | SP-A | −0.34 | 0.10 |
|  | SP-D | −0.33 | 0.10 |
|  | KL-6 | −0.34 | 0.10 |
| relative change in DLco | SP-A | −0.61 | <0.01 |
|  | SP-D | −0.42 | <0.05 |
|  | KL-6 | −0.47 | <0.05 |
| FVC = forced vital capacity; DLco = diffusing capacity of the lung for carbon monoxide; SP = surfactant protein; KL-6 = Krebs von den Lungen-6; r = correlation coefficient | | | |
