# Supplementary material for: Surfactant protein A as a biomarker of outcomes of anti-fibrotic drug therapy in patients with idiopathic pulmonary fibrosis
Source: BMC Pulm Med. 2020 Jan 31;20:27. doi: 10.1186/s12890-020-1060-y (PMC6995128; doi:10.1186/s12890-020-1060-y)
Supplement: Supplementary file 7 — Additional file 7: Table S3. Sensitivity and specificity for distinguish between the stable and the progressive patients for changes in SP-A, SP-D, and KL-6 [file 12890_2020_1060_MOESM7_ESM.docx]

| **Table S3. Sensitivity and specificity for distinguish between the stable and the progressive patients for changes in SP-A, SP-D, and KL-6.** | | | | |
| --- | --- | --- | --- | --- |
| **variable** | **cut-off** | **sensitivity** | **specificity** | **AUC** |
| **Change in SP-A in 3 months (%)** | 9.48 | 0.93 | 0.75 | 0.89 |
| **Change in SP-D in 3 months (%)** | −21.6 | 0.42 | 0.88 | 0.65 |
| **Change in KL-6 in 3 months (%)** | 12.98 | 0.94 | 0.45 | 0.72 |
| **Change in SP-A in 6 months (%)** | −0.81 | 0.81 | 0.81 | 0.89 |
| **Change in SP-D in 6 months (%)** | −12.7 | 0.53 | 0.88 | 0.73 |
| **Change in KL-6 in 6 months (%)** | −2.05 | 0.81 | 0.76 | 0.83 |
| AUC = area under the curve; SP = surfactant protein; KL-6 = Krebs von den Lungen-6 | | | | |
