# Supplementary material for: Surfactant protein A as a biomarker of outcomes of anti-fibrotic drug therapy in patients with idiopathic pulmonary fibrosis
Source: BMC Pulm Med. 2020 Jan 31;20:27. doi: 10.1186/s12890-020-1060-y (PMC6995128; doi:10.1186/s12890-020-1060-y)
Supplement: Supplementary file 8 — Additional file 8: Table S4. Baseline characteristics and clinical data of population which included patients who used corticosteroids [file 12890_2020_1060_MOESM8_ESM.docx]

| **Table S4. Baseline characteristics and clinical data of population which included patients who used corticosteroids.** | | | | |
| --- | --- | --- | --- | --- |
| **Variable** | All subjects | Stable group | Progression group | *P*-value |
|  | (n = 60) | (n = 41) | (n = 19) |  |
| **Sex M/F (n)** | 47/13 | 31/10 | 16/3 | 0.45 |
| **Age (yr)** | 70 (65–75) | 70 (66–76) | 68 (63–75) | 0.34 |
| **Smokers/never-smokers (n)** | 16/44 | 12/29 | 4/15 | 0.50 |
| **Pack-years smoking** | 32 (0.4–50) | 35 (0–48) | 26 (1.5–52) | 0.99 |
| **BMI** | 23.6 (22.1–25.8) | 23.4 (22.1–25.4) | 25.0 (21.7–26.0) | 0.40 |
| **GAP stage (n), I/II/III** | 21/29/10 | 15/19/7 | 6/10/3 | 0.90 |
| **FVC (L)** | 2.42 (2.04–2.84) | 2.38 (2.05–2.9) | 2.64 (2.03–2.73) | 0.69 |
| **% FVC (%)** | 74.7 (64.5–89.3) | 74.7 (63.8–91.7) | 75.0 (64.2–84.1) | 0.87 |
| **DLco (mL/min/mmHg)** | 10.6 (8.75–13.3) | 10.5 (8.7–13.2) | 10.7 (8.69–13.7) | 0.59 |
| **% DLco (%)** | 51.2 (43.0–57.6) | 50.5 (42.7–57.0) | 51.7 (38.4–65.3) | 0.90 |
| **PaO_2_ at rest (Torr)** | 81.5 (76.6–87.8) | 81.3 (76.5–91.2) | 83.1 (77.7–86.9) | 0.60 |
| **Minimum SpO_2_ during 6MWT (%)** | 91 (86.8–93) | 91 (87–93) | 89.5 (84.8–93) | 0.53 |
| **6MWT Distance (meter)** | 400 (360–480) | 400 (360–480) | 400 (351–485) | 0.98 |
| **SP-A (ng/mL)** | 63.3 (42.9–81.4) | 66.7 (47.9–81.0) | 50.7 (39.6–90.5) | 0.43 |
| **SP-D (ng/mL)** | 231 (163–337) | 237 (170–345) | 224 (139–302) | 0.73 |
| **KL-6 (U/mL)** | 932 (684–1475) | 941 (664–1604) | 865 (694–1260) | 0.77 |
| **pirfenidone/nintedanib** | 30/30 | 23/18 | 7/12 | 0.17 |
| **Treatment history of anti-fibrotic drug(n), yes/no** | 8/52 | 4/37 | 4/15 | 0.25 |
| Data are expressed as frequencies or medians (interquartile range). *P*-value: stable group vs. progression group. BMI = body mass index; GAP = (gender [G], age [A], and 2 lung physiology variables [P] [FVC and DLco]); FVC = forced vital capacity; DLco = diffusing capacity of the lung for carbon monoxide; PaO_2_ = partial pressure of arterial oxygen; SpO_2_ = arterial oxygen saturation measured by pulse oximetry; 6MWT = 6 minute-walk test; SP = surfactant protein; KL-6 = Krebs von den Lungen-6 | | | | |
