# Supplementary material for: Surfactant protein A as a biomarker of outcomes of anti-fibrotic drug therapy in patients with idiopathic pulmonary fibrosis
Source: BMC Pulm Med. 2020 Jan 31;20:27. doi: 10.1186/s12890-020-1060-y (PMC6995128; doi:10.1186/s12890-020-1060-y)
Supplement: Supplementary file 9 — Additional file 9: Table S5. Serum levels of SP-A, SP-D, and KL-6 at baseline, 3, and 6 months of population which included patients who used corticosteroids [file 12890_2020_1060_MOESM9_ESM.docx]

| **Table S5. Serum levels of SP-A, SP-D, and KL-6 at baseline, 3, and 6 months of population which included patients who used corticosteroids.** | | | |
| --- | --- | --- | --- |
|  | Baseline | 3 months | 6 months |
| **Stable group** |  |  |  |
| **SP-A (ng/mL)** | 66.7 (47.9–81.0) | 54.1 (38.4–82.6) * | 56.4 (37.9–74.4) * |
| **SP-D (ng/mL)** | 237 (170–345) | 190 (142–315) * | 185 (128–265) * |
| **KL-6 (U/mL)** | 941 (664–1604) | 955 (600–1250) | 887 (591–1352) * |
| **Progression group** |  |  |  |
| **SP-A (ng/mL)** | 50.7 (39.6–90.5) | 61.9 (43.1–107.3) * | 64.3 (38.7–105.3) * |
| **SP-D (ng/mL)** | 224 (139–302) | 232 (178–302) | 221 (168–344) |
| **KL-6 (U/mL)** | 865 (694–1260) | 1014 (613–1742) | 1139 (635–1509) |
| Data are expressed as medians (interquartile range). | | | |
| *: Bonferroni-corrected p < 0.05 baseline vs. 3 or 6 months. ^#^: p < 0.05 stable group vs. progression group. SP = surfactant protein; KL-6 = Krebs von den Lungen-6 | | | |
