# Supplementary material for: Surfactant protein A as a biomarker of outcomes of anti-fibrotic drug therapy in patients with idiopathic pulmonary fibrosis
Source: BMC Pulm Med. 2020 Jan 31;20:27. doi: 10.1186/s12890-020-1060-y (PMC6995128; doi:10.1186/s12890-020-1060-y)
Supplement: Supplementary file 10 — Additional file 10: Table S6. Prediction of stability at 6 months from administration of anti-fibrotic drugs in univariate analysis of population which included patients who used corticosteroids [file 12890_2020_1060_MOESM10_ESM.docx]

| **Table S6. Prediction of stability at 6 months from administration of anti-fibrotic drugs in univariate analysis** **of population which included patients who used corticosteroids.** | | |
| --- | --- | --- |
| **Variable** | OR (95% CI) | *P*-value |
| **Sex, Male** | 0.58 (0.12–2.22) | 0.44 |
| **Age** | 1.05 (0.97–1.14) | 0.21 |
| **Smokers** | 1.55 (0.43–5.65) | 0.50 |
| **Pack-years smoking** | 1.00 (0.98–1.02) | 0.97 |
| **BMI** | 0.90 (0.75–1.05) | 0.19 |
| **% FVC (%)** | 1.00 (0.98–1.04) | 0.76 |
| **% DLco (%)** | 0.99 (0.95–1.03) | 0.68 |
| **PaO_2_ at rest (Torr)** | 1.02 (0.97–1.08) | 0.45 |
| **Minimum SpO_2_ during 6MWT (%)** | 1.06 (0.93–1.20) | 0.38 |
| **6MWT Distance (m)** | 1.00 (0.99–1.00) | 0.97 |
| **SP-A (ng/mL)** | 1.00 (0.99–1.01) | 0.93 |
| **SP-D (ng/mL)** | 1.00 (1.00–1.01) | 0.44 |
| **KL-6 (U/mL)** | 1.00 (1.00–1.00) | 0.99 |
| **Pirfenidone** | 2.19 (0.72–6.70) | 0.16 |
| **Treatment history of anti-fibrotic drug** | 0.42 (0.09–1.89) | 0.26 |
| **Change in SP-A in 3 months (%)** | 0.90 (0.84–0.94) | <0.01 |
| **Change in SP-D in 3 months (%)** | 0.98 (0.97–1.00) | <0.05 |
| **Change in KL-6 in 3 months (%)** | 0.96 (0.92–0.99) | <0.01 |
| **Change in SP-A in 6 months (%)** | 0.90 (0.84–0.94) | <0.01 |
| **Change in SP-D in 6 months (%)** | 0.96 (0.93–0.99) | <0.01 |
| **Change in KL-6 in 6 months (%)** | 0.93 (0.89–0.96) | <0.01 |
| OR = odd’s ratio; BMI = body mass index; FVC = forced vital capacity; DLco = diffusing capacity of the lung for carbon monoxide; PaO_2_ = partial pressure of arterial oxygen; SpO_2_ = arterial oxygen saturation measured by pulse oximetry; 6MWT = 6 minute-walk test; SP = surfactant protein; KL-6 = Krebs von den Lungen-6 | | |
