# Supplementary material for: Surfactant protein A as a biomarker of outcomes of anti-fibrotic drug therapy in patients with idiopathic pulmonary fibrosis
Source: BMC Pulm Med. 2020 Jan 31;20:27. doi: 10.1186/s12890-020-1060-y (PMC6995128; doi:10.1186/s12890-020-1060-y)
Supplement: Supplementary file 11 — Additional file 11: Table S7. Prediction of stability at 6 months from administration of anti-fibrotic drugs in multivariate analysis of population which included patients who used corticosteroids [file 12890_2020_1060_MOESM11_ESM.docx]

| **Table S7. Prediction of stability at 6 months from administration of anti-fibrotic drugs in multivariate analysis** **of population which included patients who used corticosteroids.** | | | | | |  |
| --- | --- | --- | --- | --- | --- | --- |
|  | model 1 | | model 2 | | model 3 (model 1 + model 2) | |
| **variable** | OR (95% CI) | *P*-value | OR (95% CI) | *P*-value | OR (95% CI) | *P*-value |
| **Change in SP-A in 3 months (%)** | 0.88 (0.80–0.94) | <0.01 | − | − | 0.90 (0.80–0.98) | <0.05 |
| **Change in SP-D in 3 months (%)** | 1.02 (0.99–1.06) | 0.12 | − | − | 1.03 (1.00–1.08) | 0.05 |
| **Change in KL-6 in 3 months (%)** | 0.99 (0.94–1.02) | 0.57 | − | − | 0.99 (0.93–1.03) | 0.74 |
| **Change in SP-A in 6 months (%)** | − | − | 0.91 (0.84–0.96) | <0.01 | 0.91 (0.83–0.97) | <0.01 |
| **Change in SP-D in 6 months (%)** | − | − | 1.00 (0.96–1.03) | 0.83 | 1.00 (0.95–1.04) | 0.90 |
| **Change in KL-6 in 6 months (%)** | − | − | 0.95 (0.89–1.00) | <0.05 | 0.96 (0.89–1.03) | 0.25 |
| OR = odd’s ratio; SP = surfactant protein; KL-6 = Krebs von den Lungen-6 | | | | | | |
